# Continuous interstitial glucose monitoring in diabetic and non-diabetic critically ill patients is simple and accurate: comparison with venous, arterial and capillary glucose measurements

**DOI:** 10.1007/s00592-025-02531-1

**Published:** 2025-06-07

**Authors:** Davide Chiumello, Mattia Passeri, Silvia Coppola, Elena Chiodaroli, Simone Carnier, Marialaura Montante, Tommaso Pozzi, Ilaria Goggi, Francesco Bifari, Umberto Mortola, Lucia Centofanti, Franco Folli

**Affiliations:** 1 Department of Anesthesia and Intensive Care, ASST Santi Paolo e Carlo, San Paolo University Hospital, Via Di Rudini 8, Milan, Italy; 2 Department of Health Sciences, University of Milan, Milan, Italy; 3 Coordinated Research Center on Respiratory Failure, University of Milan, Milan, Italy; 4 Laboratory of Cell Metabolism and Regenerative Medicine, Department of Medical Biotechnology and Translational Medicine, University of Milan, LITA, Segrate, Italy; 5 Departmental Unit of Diabetes and Metabolic Disorders, ASST Santi Paolo e Carlo, San Paolo University Hospital, Via Di Rudini 8, Milan, Italy

**Keywords:** Continuous glucose monitoring, Diabetes, Intensive care unit, Venous glycemia, Arterial glycemia, Capillary glycemia

## Abstract

**Introduction:**

To reduce mortality, thigh glycemic control is recommended in critically ill patients due to their extreme glycemic variability. Continuous glucose monitoring (CGM) devices allows frequent determination of blood glucose levels; however, conflicting results have been reported from studies assessing their accuracy in critically ill patients. Aim of this study was to assess the repeatability and the analytical and clinical accuracy of FreeStyle Libre 2 (FSL-)CGM.

**Materials and methods:**

Prospective single-center observational study enrolling 40 critically ill patients. For four consecutive days, we measured three consecutive interstitial FSL-CGM-derived glucose levels, along with one arterial and venous blood gas analysis and a capillary-derived blood glucose level, obtaining a total of 480 FSL-CGM-derived glucose measurements and 160 measurements from arterial and venous blood gas analysis and from capillary glucose.

**Results:**

The mean blood glucose levels in the three daily timepoints from FSL-CGM were 130 ± 35, 131 ± 35 and 131 ± 35 mg/dL (*p* = 0.660). The Bland-Altman analysis comparing arterial BGA- and FSL-CGM-derived blood glucose levels had a bias of 10.3 mg/dL with limits of agreement from − 27.2 to 47.7. The mean absolute relative difference (MARD) between FSL-CGM and arterial blood gas analysis was 12 ± 10%. The Clarke, Parkes and Surveillance error grid analyses comparing arterial BGA- and FSL-CGM-derived blood glucose levels showed a good clinical accuracy. The presence of diabetes did not influence analytical accuracy, while the use of vasopressors was associated with a higher MARD.

**Conclusions:**

FSL-CGM demonstrated reproducibility and reliable analytical and clinical accuracy in critically ill patients, without difference between diabetic and non-diabetic patients, over a period of up to 96 h (4 days).

**Supplementary Information:**

The online version contains supplementary material available at 10.1007/s00592-025-02531-1.

## Introduction

Critically ill patients are characterized by an extreme glycemic variability, ranging from hypoglycemia to hyperglycemia, due to their specific pathological conditions and exogenous factors, such as proinflammatory cytokines, stress, and increased hormone levels [1–4]. Both glycemic variability and poor glycemic control have been associated with increased morbidity and mortality [3, 5–9]. Therefore, personalized glycemic control should be implemented in daily clinical practice [10].

To reduce mortality, recent guidelines and expert opinions recommend glycemic control with an upper blood glucose limit of 180 mg/dL in order to avoid hyperglycemia, and to prevent hypoglycemia [11–14]. Extremely frequent blood glucose measurements, ranging from every 30 to 120 min, are advised for the safe adjustment of intravenous insulin therapy [15, 16].

Arterial or venous blood gas analyses (BGAs) and laboratory measurements are routinely used to assess blood glucose levels [17]. However, these techniques can provide only intermittent assessments, potentially missing significant glycemic fluctuations, while further increasing an already high nurse workload [12, 18]. In the daily intensive care clinical practice, blood glucose is typically measured by BGA using arterial or venous samples [19–21].

Continuous glucose monitoring systems (CGMs) have been developed over the years [2, 20, 22–26]. These devices use intravascular or subcutaneous catheters to continuously measure glucose levels without requiring external analyses of arterial or venous samples [2]. Over the last decades, CGMs have demonstrated to improve glucose control by reducing both hyperglycemic and hypoglycemic events [27–29]. Despite these benefits, CGMs are not yet widely adopted in critically ill patients [2].

Among available CGMs, the FreeStyle Libre 2 (FSL-CGM, Abbott Diabetes Care, Alameda, CA, USA) monitoring system is a non-invasive device approved for monitoring subcutaneous glucose concentration in both outpatient and inpatient settings [30, 31]. In non-critically ill patients, FSL-CGM has shown good accuracy compared to capillary blood glucose monitoring and other CGMs [27, 32, 33]. However, studies in critically ill patients evaluating the accuracy of FSL-CGM compared to arterial or venous laboratory or gas analysis measurements have yielded conflicting results, mainly due to small sample sizes, retrospective study designs, and inconsistent reference methods [34–38]. Additionally, critically ill patients often experience variable stress responses, complex medical conditions, catecholamine infusion, and fluctuating nutritional support, further complicating the application of CGMs. The purpose of this study is to compare glycemic monitoring by FreeStyle Libre 2 system to three different commonly employed methods in ICU during critical illness. The main endpoints of our study are: (1) to assess the repeatability of the FSL-CGM; (2) to evaluate the accuracy of the FSL-CGM by comparing its readings with reference glucose measurements, including arterial and venous blood gas analysis and capillary blood glucose measurements; (3) to explore factors influencing FSL-CGM accuracy.

## Materials and methods

### Study design

This was a prospective single-center observational study. The Institutional Review Board of ASST Santi Paolo e Carlo approved the study, and written informed consent was obtained according to Italian regulations (2022/ST/188).

### Population

All patients admitted to the ICU with an arterial catheter and a central venous catheter were considered eligible for the study. The only exclusion criterion was an estimated ICU stay of less than four days.

### Study protocol and data collection

At ICU admission, demographic and anthropometric variables were obtained and recorded, as well as history of diabetes. After enrollment, each patient had a FreeStyle Libre 2 sensor applied to the back of one arm, as prescribed by the manufacturer. The glucose sensor was placed on the skin of external lateral-posterior portion of the left arm, 3–4 cm below the lower extremity of the deltoid muscle. In this position, the glucose sensor is easily accessible by nurses, skin is well perfused and contact pressure is minimal or absent. For each patient the following measurements were taken in the same time frame: (a) three consecutive glucose measurements with FSL-CGM at 60-second intervals each; (b) one arterial blood gas analysis; (c) one venous blood gas analysis; and (d) one capillary glucose measurement. These measurements were repeated for four consecutive days, thus obtaining a total of 480 FSL-CGM-derived measurements and 160 measurements for arterial, venous BGA and capillary glucose. Moreover, the use of exogenous catecholamines and doses, the daily fluid balance, insulin use, SOFA score, hemoglobin levels, and ventilation modes (invasive vs. non-invasive) were recorded daily.

## Statystical analysis

### Sample size

We estimated a mean glucose of 126 ± 35 mg/dL; repeating the measurements three times, with an effect size of 0.265 [2]. Assuming an identical distribution of FSL-CGM-derived measurements, we hypothesized that a sample of 40 patients, with each patient measured for four consecutive days for a total of 160 measurements, would provide the study power of 0.90 with an alpha of 0.05 in assessing repeatability.

### Repeatability

FSL-CGM-derived measurements were repeated three times at one-minute intervals each day for each patient. To assess repeatability, differences among interstitial glucose measurements obtained in the three daily timepoints within each patient were analyzed using one-way ANalysis Of VAriance [39] or Friedman Test, as appropriate.

### Accuracy

To assess accuracy, the average of the three values measured at different timepoints by FSL-CGM was used, given the repeatability of the method.

For analytical accuracy, comparing FSL-CGM-derived measurements and measurements obtained with the arterial and venous BGA and capillary glucose (assumed as gold standard methods), three methods were used: (1) Bland-Altman analysis; (2) computation of Mean Absolute Relative Difference (MARD) between interstitial measurements and the three reference methods [40]; (3) analysis according to Finfer et al. [21], checking that 98% of the data pairs between FSL-CGM-derived measurements and each of the reference method must have a percentage difference between − 12.5% and 12.5% for blood glucose values greater than 100 mg/dl, or a difference between − 10 and 10 mg/dL for blood glucose values below 100 mg/dl.

For clinical accuracy, three error grids, with different thresholds and limits, were used: Clarke Error Grid [41], Parkes Error Grid [42], and Surveillance Error Grid [43].

### Subpopulation analysis

To evaluate the influence clinical covariates on FSL-CGM accuracy, we calculated and compared MARDs, and also constructed Bland-Altman plots for the comparison between arterial blood gas analysis only and FSL-CGM across two patient subpopulations: presence of diabetes upon ICU admission and use of vasopressors.

Data are expressed as mean ± SD or as median [interquartile range], as appropriate (Shapiro-Wilk’s test). Statistical analysis was performed using RStudio version 2023.6.1.5242023.6.1.524 (Posit team (2023). RStudio: Integrated Development Environment for R. Posit Software, PBC, Boston, MA).

## Results

### Study population

Forty patients were enrolled, of whom 28 were men (70%) and 12 were women (30%) with an age of 64 [57–72] years, a BMI of 26 [20, 23–28] kg/m^2^. Thirty-two (80%) patients were non-diabetic and 8 (20%) had diabetes (Table 1). On the first measurement day, average blood glucose levels were 140 ± 32 mg/dL.

**Table 1:** Baseline characteristics of the study population

	*n* = 40
Age, *years*	67 [57–72]
Male sex, *% (n)*	70 (28)
Body mass index, *kg/m*^*2*^	26 [23–29]
SOFA score ad ICU admission	6 [3–7]
History of diabetes, *% (n)*	21 (8)
Insulin use, *% (n)*	33 (13)
Vasopressor use, *% (n)*	45 (18)

### Repeatability

**Fig. 1:**
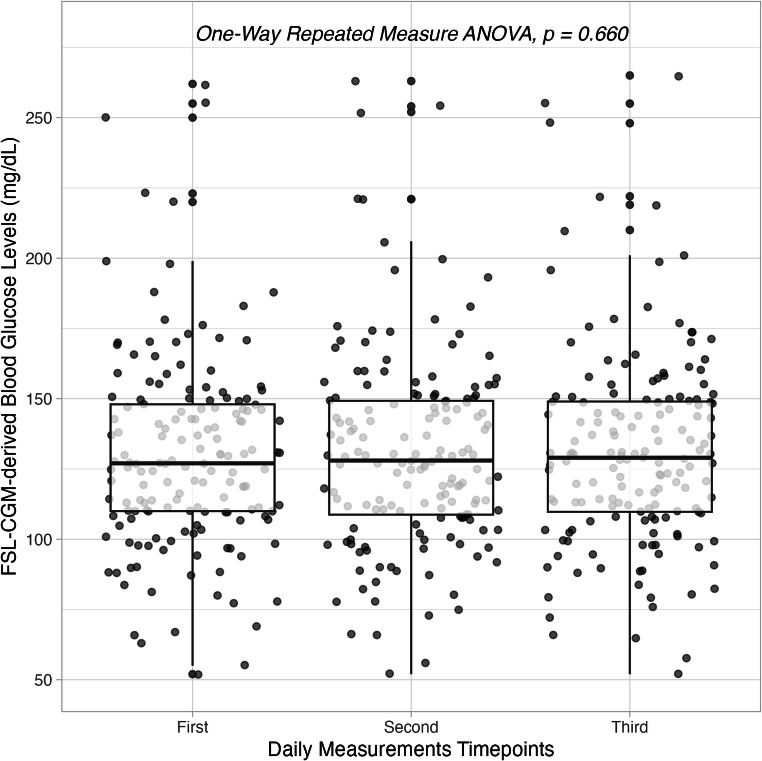
Repeatability of FreeStyle Libre (FSL-CGM)-derived blood glucose levels in the three daily measurements timepoints. *ANOVA* analysis of variance

The mean blood glucose levels in the three daily timepoints from FSL-CGM were 130 ± 35, 131 ± 35 and 131 ± 35 mg/dL, respectively. No statistically significant difference among the three daily timepoints was found (*p* = 0.660) (Fig. 1).

### Analytical accuracy

**Fig. 2:**
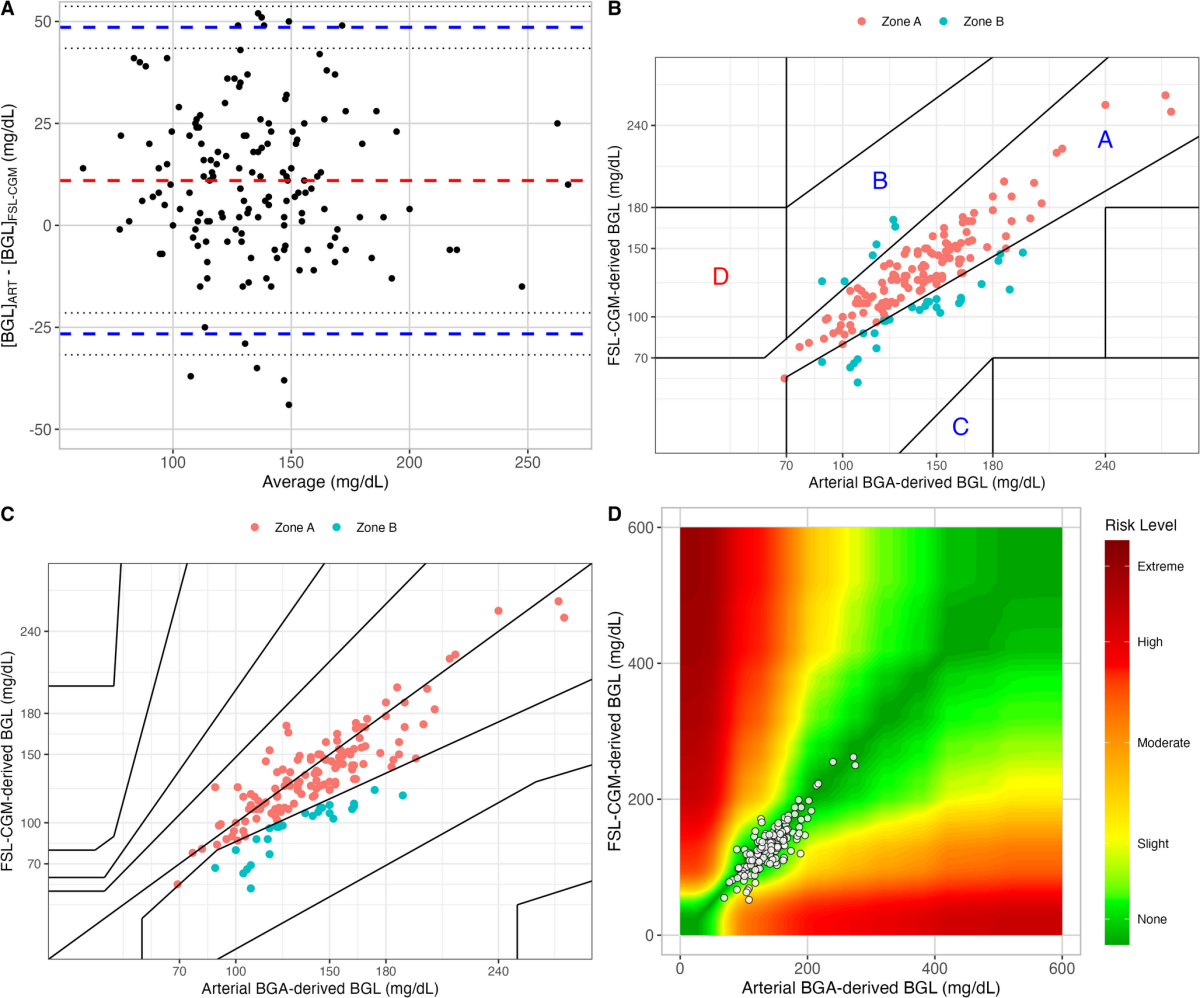
Analytical and clinical accuracy of FreeStyle Libre Continuous Glucose Monitoring System (FSL-CGM) and arterial blood gas (ART) in determining blood glucose levels (BGL). **A**: Bland-Altman plot. Red dashed line represents the mean difference (bias), while blue dashed lines represent the lower and the upper limits of agreement (LOAs). Dotted lines represent confidence intervals. **B**: Clark Error Grid. Red points fall in Zone A, while blue points fall in Zone B. **C**: Parkes Error Grid. Red points fall in Zone A, while blue points fall in Zone B. **D**: Surveillance Error Grid. The color gradient from green to red represents an increasing level of risk of bias

The Bland-Altman analysis comparing arterial BGA- and FSL-CGM-derived blood glucose levels had a bias of 10.3 mg/dL with limits of agreement from − 27.16 to 47.7 (Fig. 2 Panel A). The Bland-Altman comparing venous BGA-derived and capillary blood glucose levels with FSL-CGM-derived blood glucose levels presented similar bias and limits of agreement (Figure S1).

The MARD between FSL-CGM and arterial BGA was 12 ± 10%, between FSL-CGM and venous BGA was 11 ± 11%, and between FSL-CGM and capillary glucose was 11 ± 10%.

The agreement analysis according to Finfer et al. revealed that 61 and 69% of the measurement pair from FSL-CGM and arterial blood gas analysis greater or lower than 100 mg/dL, respectively, exhibited permissive percentage differences. Data from venous blood gas analysis and capillary glucose measurements presented similar percentages.

Table 2 summarizes the analytical accuracy data.

**Table 2:** Analytical accuracy between freestyle libre (FSL-CGM) and arterial or venous blood gas analyses (BGAs) or capillary glucose measurements according to Bland-Altman, MARD or Finfer’s analyses. *LoAs* limits of agreement; *MARD* mean absolute relative difference, *SD* standard deviation

	FSL-CGM vs. Arterial BGA	FSL-CGM vs. Venous BGA	FSL-CGM vs. Capillary Glucose
Bias, *mg/dL*	10	9	2
Lower and upper LoAs, *mg/dL*	-27; 48	-28; 46	-37; 42
MARD ± SD, *%*	12 ± 10	12 ± 11	11 ± 11
Finfer’s standard for blood glucose levels			
> 100 mg/dL, *%*	61	66	70
≤ 100 mg/dL, *%*	69	45	71

### Clinical accuracy

The Clarke error grid analysis comparing arterial BGA- and FSL-CGM-derived blood glucose levels showed a good clinical accuracy, with all of the measurement falling in zone A (*n* = 125, 78%) and B (*n* = 35, 22%) (Fig. 2 Panel B). Also in the Parkes Error Grid, all of the measurements fell in zone A (*n* = 122, 76%) and B (*n* = 38, 24%). According to the Surveillance Error Grid, 84.4% (*n* = 135) of the values were in a no-risk zone, 15% (*n* = 24) in a low-risk zone, and only one value (0.6%) in a moderate-risk zone, in the low range. Similar analysis comparing the venous blood gas analysis, capillary blood glucose levels and FSL-CGM are presented in the Supplementary Material (Figure S2-S3).

### Subpopulation analysis

Stratifying the population according to the presence of diabetes showed similar MARDs (*p* = 0.314) and Bland-Altman plots; (Table S1−S2, Figure S3). The use of vasopressors was associated with a higher MARD (*p* = 0.010).

## Discussion

The major findings of this study, which evaluated the performance of the FSL-CGM in critically ill patients, were: (1) FSL-CGM provided repeatable measurements in this population; (2) the agreement between the FSL-CGM and the three reference methods (arterial and venous BGA and capillary glucose) was clinically acceptable and similar among these methods and (3) the FSL-CGM accuracy was not affected by the presence of diabetes, while the use of vasopressors was associated with a lower analytical accuracy.

Critically ill patients are often characterized by significant glycemic variability due to underlying disease, proinflammatory status, steroids use, and parenteral nutrition [44]. Poor glycemic control is associated with increased morbidity and mortality [7, 45]. Therefore, closely monitoring of blood glucose levels is essential for the early detection of hypo- and hyper-glycemic events, to potentially reduce mortality rates [2].

In the hospital clinical settings, gold standards for blood glucose measurements are venous or arterial determinations in clinical laboratory by the glucose oxidase method. Alternatively, blood glucose can be measured via point-of-care methods, such as a gas analyzer, which have demonstrated excellent accuracy and performance in critically ill patients [20].

However, these methods are quite time-consuming, intermittent, and may fail to capture severe hypo- or hyperglycemic events, due to the lack of clinical signs in the majority of critically ill patients [2].

Over the past decades, several CGMs have been developed to enable continuous monitoring of glucose levels through subcutaneous or intravascular catheters (invasive or non-invasive techniques). These systems offer the advantage of reducing costs and nurse workload while providing more comprehensive glucose data [2]. At the present time the CGMs are still not often applied in critically ill patients [36].

Bolinder et al. demonstrated that CGM used in patients with type 1 diabetes significantly reduced hypoglycemia time by up to 38% and decreased the frequency of hypoglycemic events. In non-critically ill patients, CGM use not only improved glycemic control by reducing hypoglycemic events, but also enhanced quality of life [46, 47].

Among the various CGMs, the FreeStyle monitoring system utilizes a small subcutaneous sensor measuring interstitial glucose concentrations by the glucose oxidase method. This glucose monitoring technology is widely employed in clinical practice of patients with type 1 diabetes, type 2 diabetes and gestational diabetes for hospitalized and ambulatory patients.

As previously shown in non-critically ill patients, FSL-CGM has demonstrated to be repeatable even in our study population conditions, in which micro-circulatory disfunction could possibly be present, altering the physiological relationship between intravascular and interstitial fluid [48].

Our study, using Bland-Altman analysis, demonstrated that FSL-CGM detected slightly lower glucose values compared to arterial, venous and capillary blood glucose analyses. The mean difference between glucose values detected in the interstitial compartment by FSL-CGM and in arterial blood, around 10 mg/dL, could be ascribed to physiological differences between arterial and tissue glucose concentrations, which continuously extract glucose from blood supply. Accordingly, we found slightly lower biases for venous and capillary vs. interstitial glucose measurement comparisons, possibly reflecting the physiological smaller glucose concentration gradient between capillary or interstitial compartment with respect to tissues. Similarly, prior findings in critically ill patients reported slightly lower FSL-CGM-derived glucose levels than reference methods [35, 37].

We additionally used the MARD analysis, which provides an analytical comparison between two glucose measurement methods: for being clinically acceptable, the MARD should be lower than 14%, while values higher than 18% are considered not acceptable [49]. We found acceptable and similar MARD values for all comparisons.

Finally, as glucose measurement errors could directly influence the therapeutic strategy, Clarke, Parkes and Surveillance error grid analyses have been applied in order to assess clinically-relevant accuracy in glucose measurements between the CMGs and reference method [2]. Essentially, all three methods classify each measurement pair bias according to the clinical risk of a therapeutic misconduct, visually representing them in risk zones [49].

The clinical accuracy was acceptable for both the Clarke and Parkes error grids when comparing arterial and venous vs. interstitial glucose measurements; indeed, all measurements fell into zone A or B, with no risk of inappropriate treatment or effect on clinical outcome. Only one capillary vs. interstitial measurement pair fell into Clarke error grid zone D (0.6%), potentially leading to an untreated hypoglycemia. The Surveillance error grid, which represents clinical risk as a continuous spectrum due to the high number of risk zones, led to similar results, being all measurements pair associated with at most moderate clinical risk.

We hypothesized that the presence of diabetes or the use of vasopressors could alter the physiological relationship between blood and the interstitial fluid. Ancona et al. found in critically ill patients with diabetes a MARD of 14% comparing the FreeStyle and the arterial blood gas analysis [35]. Accordingly, our results showed that the presence of diabetes had no influence on the analytical accuracy of FSL-CGM. Given the highly similar glucose values obtained in venous, arterial, capillary and subcutaneous interstitial tissue in both diabetic and non-diabetic patients, we are quite confident that insulin administration in diabetic patients did not impact interstitial *vs*. blood glucose dynamics.” In critically ill patients with hyperglycemia the FreeStyle has a MARD of 18% that was considered still acceptable in clinical management [34]. On the contrary a retrospective study in critically ill patients comparing the accuracy of the FreeStyle monitoring and the arterial blood glucose although found a MARD of 13% for all paired values, presented a significantly higher MARD in the hypoglycemic and hyperglycemic (32% and 18% respectively) [36]. These negative data could be explained by the relatively large number of devices which presented a failure (12%) and the high variable time intervals between the FreeStyle and the arterial blood analysis.

Ultimately, we found that the use of vasopressor is associated with a lower analytical accuracy, possibly due to the disruption of the physiological relationship between microcirculation and interstitial fluid. One of the major strengths of this study was the evaluation of the FreeStyle Libre 2 CGM in critically ill patients continuously for up to four days, compared with other three standard methods used for glucose measurement using arterial, venous and capillary blood. Our study has some limitations: (1) the observational and single-center nature of the study; (2) the relatively small sample size, which could add unexplained variability due to the inflation by the repetition of measurements in subsequent days; (3) the sensor utilization for only 96 h (4 days) may not capture the long term sensor behaviour, because these sensors have been originally developed to be utilized for a total of 14 consecutive days. The reason why we decided to use the sensor for 96 h (which is only 30% of the expected utilization time of the sensor) is that the length of stay in the ICU is unpredictable for the severity of clinical conditions, and we wanted to be almost certain to capture the same length of utilization in most if not all patients studied. Future studies will be able to address the reliability of long term sensor behavior in the ICU; (4) in this study we wanted to compare the reliability of interstitial glucose measurements with standard capillary, venous and arterial blood glucose measurements. Because we did not know the reliability of interstitial glucose measurements in critically ill patients, clinical decision making/insulin treatment adjustments were made on the basis of capillary and venous glucose values.

In conclusion, on the basis of our current study and previous studies, we believe that CGM readings could be considered reliable glucose measurements in the near future, to be employed in the Intensive Care Unit, for clinical decision making and/or insulin therapy adjustment in real time.(36–38).

## Conclusions

Although the FreeStyle Libre 2 is defined as a continuous glucose monitoring device, it is a minimally invasive automatic intermittent monitoring system which analyzes the subcutaneous glucose every 15 min. Our data suggest that FSL-CGM demonstrates repeatability and acceptable analytical and clinical accuracy in critically ill patients, without difference between diabetic and non-diabetic, over a period of up to 96 h. Thus, the use of the FSL-CGM system could improve glycemic control, possibly leading to a better outcome, while reducing nurse workload and healthcare costs, particularly in critical patients which may experience high glycemic variability also secondary to multiple drugs employed. Larger and longer clinical studies could be implemented to address this possibility.

## Electronic supplementary material

Below is the link to the electronic supplementary material.

Supplementary Material 1.
