# Supplementary material for: Continuous interstitial glucose monitoring in diabetic and non-diabetic critically ill patients is simple and accurate: comparison with venous, arterial and capillary glucose measurements
Source: Acta Diabetol. 2025 Jun 7;62(8):1173–81. doi: 10.1007/s00592-025-02531-1 (PMC12364967; doi:10.1007/s00592-025-02531-1)
Supplement: Supplementary file 1 — Supplementary Material 1. [file 592_2025_2531_MOESM1_ESM.docx]

**Continuous interstitial glucose monitoring in diabetic and non-diabetic critically ill patients is simple and accurate. Comparison with venous, arterial and capillary glucose measurements.**

*Supplementary Digital Material*

***Additional Results***


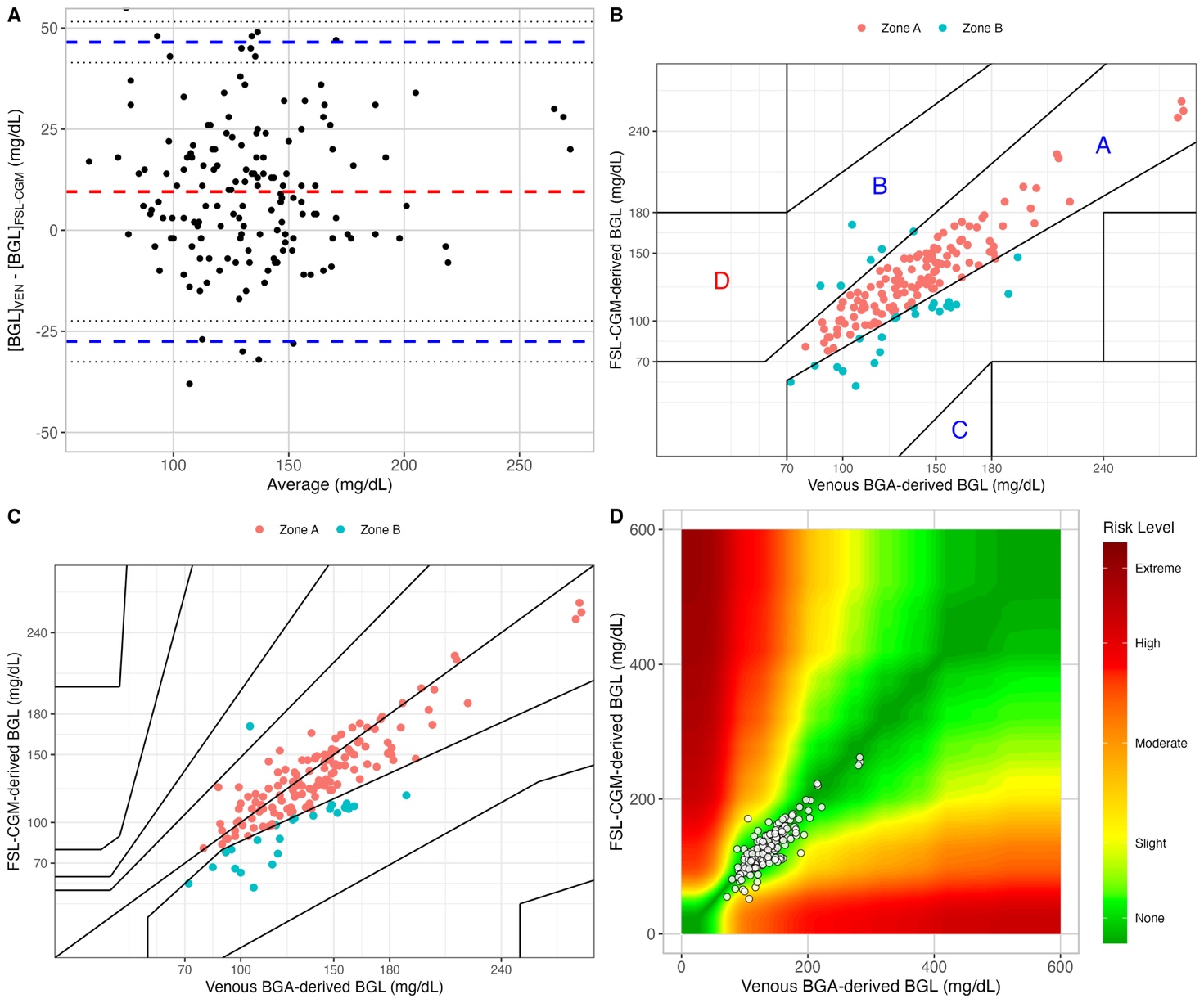


***Figure S1****: Analytical and clinical accuracy of FreeStyle Libre Continuous Glucose Monitoring System (FSL-CGM) and venous blood gas analysis (VEN) in determining blood glucose levels (BGL). A: Bland-Altman plot. Red dashed line represents the mean difference (bias), while blue dashed lines represent the lower and the upper limits of agreement (LOAs). Dotted lines represent confidence intervals. B: Clark Error Grid. Red points fall in Zone A, while blue points fall in Zone B. C: Parkes Error Grid. Red points fall in Zone A, while blue points fall in Zone B. D: Surveillance Error Grid. The color gradient from green to red represents an increasing level of risk of bias.*


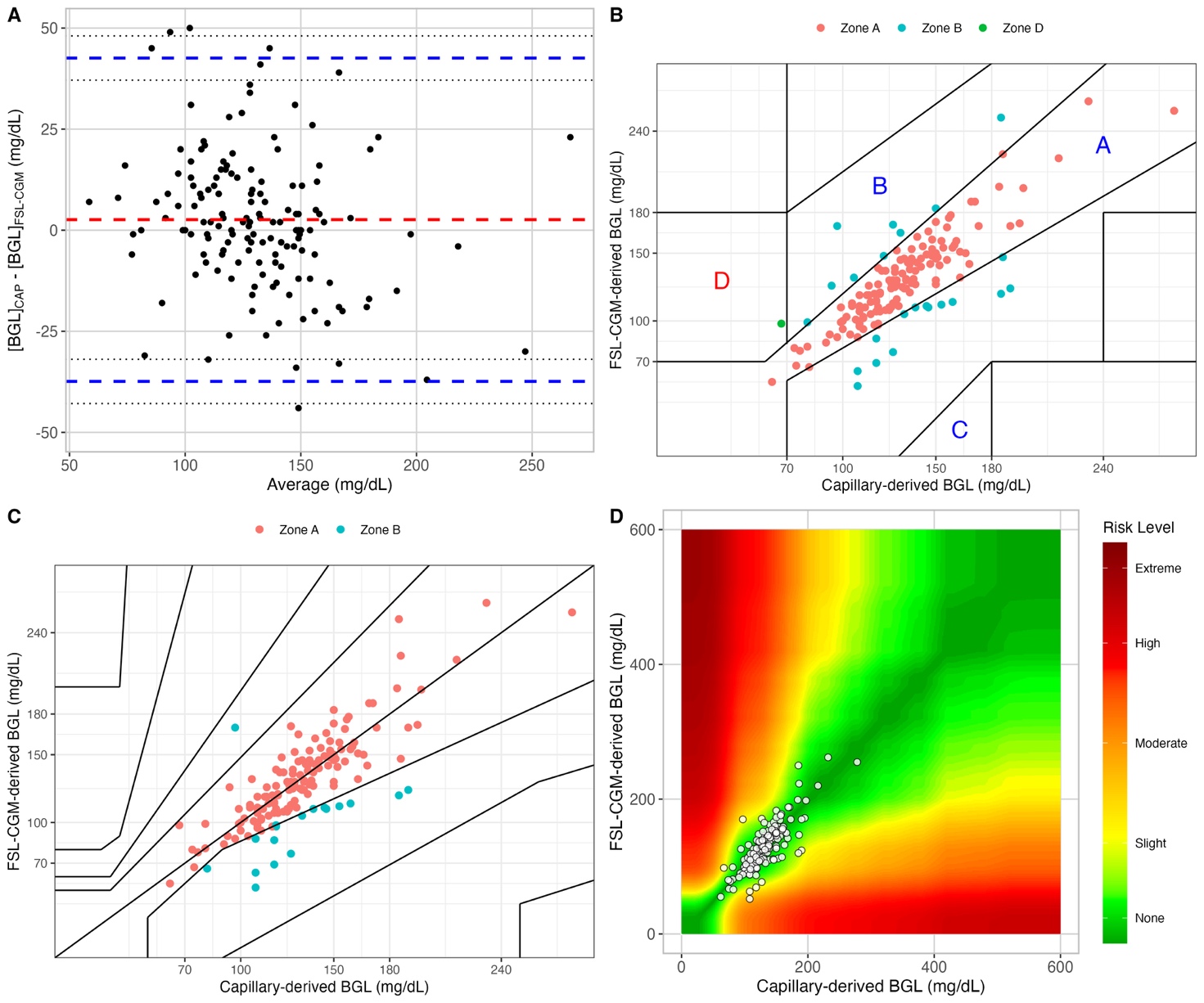


***Figure S2****: Analytical and clinical accuracy of FreeStyle Libre Continuous Glucose Monitoring System (FSL-CGM) vs capillary glucose (CAP) in determining blood glucose levels (BGL). A: Bland-Altman plot. Red dashed line represents the mean difference (bias), while blue dashed lines represent the lower and the upper limits of agreement (LOAs). Dotted lines represent confidence intervals. B: Clark Error Grid. Red points fall in Zone A, while blue points fall in Zone B. C: Parkes Error Grid. Red points fall in Zone A, while blue points fall in Zone B. D: Surveillance Error Grid. The color gradient from green to red represents an increasing level of risk of bias.*

|  | Patients with diabetes  (n = 8) | Patients without diabetes  (n = 32) | *p* |
| --- | --- | --- | --- |
| MARD ± SD | 13.7 ± 10.7 | 11.6 ± 10.1 | *0.314* |
| Bias | 4.7 | 7.2 | - |
| Limits of Agreement (lower; upper) | -61.5; 70.8 | -35.5; 49.9 | - |

***Table S1.*** *Mean absolute relative difference (MARD) and Bland-Altman bias and limits of agreement in patients with or without diabetes.*

|  | Use of vasopressors  (n = 63) | No use of vasopressors  (n = 97) | *p* |
| --- | --- | --- | --- |
| MARD ± SD | 14.8 ± 11.5 | 10.1 ± 8.9 | ***0.010*** |
| Bias | 3.2 | 10.8 | - |
| Limits of Agreement (lower; upper) | -37.6; 43.9 | -40.5; 62.1 | - |

***Table S2.*** *Mean absolute relative difference (MARD) and Bland-Altman bias and limits of agreement according to the use of vasopressors.*

**
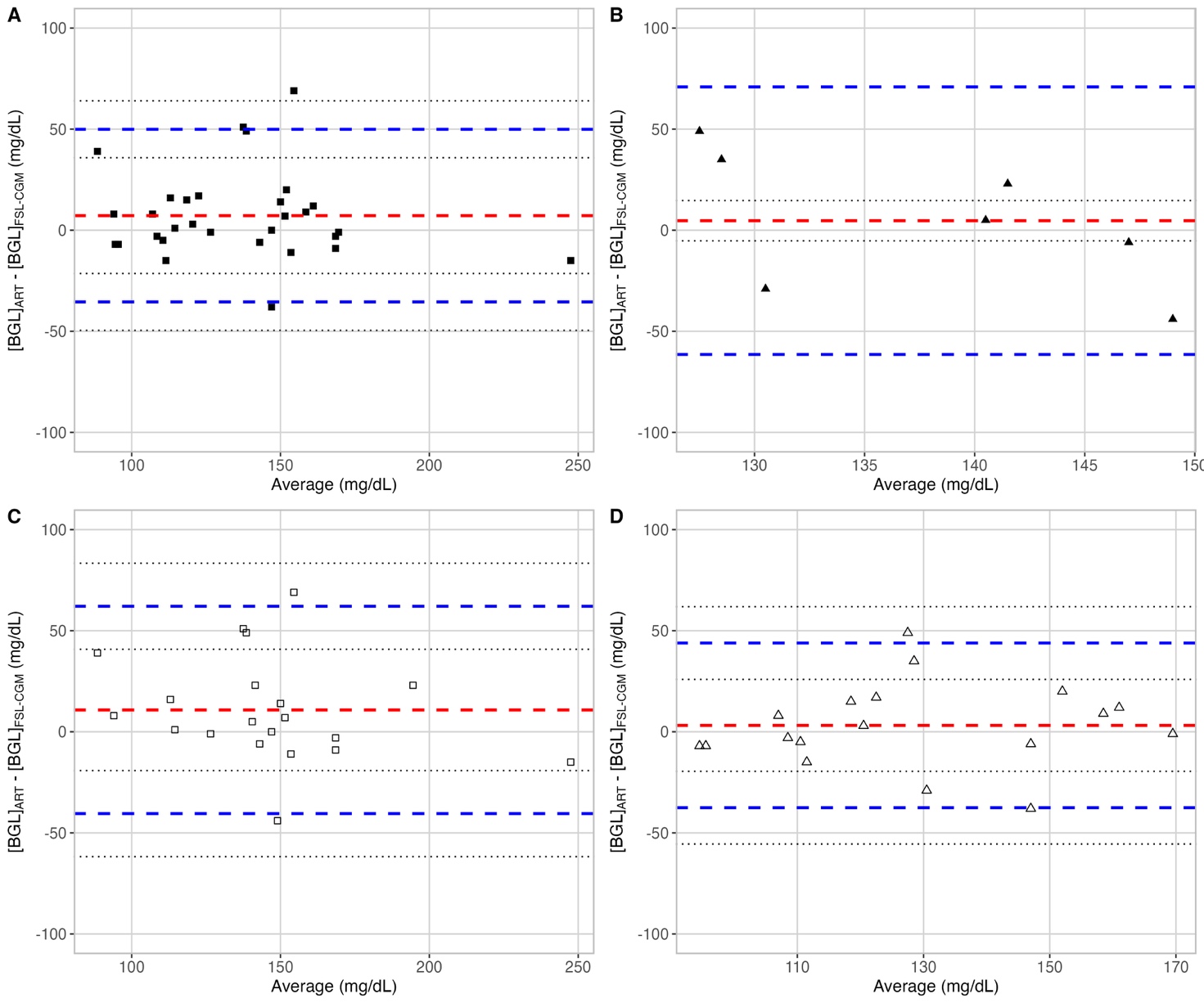
**

***Figure S3****. Analytical accuracy in patients with or without diabetes (A, B) and the use or absence of any vasopressor support in the study period (C, D) for FreeStyle Libre Continuous Glucose Monitoring System (FSL-CGM) vs arterial blood gas analysis (ART). Figure A, black squares: patients without diabetes; figure B, black triangles: patients with diabetes. Figure C, white squares: patients without vasopressor support during the study period; Figure D: patients with vasopressor support during the study period.*
